# Beta Cell Function as a Baseline Predictor of Weight Loss After Bariatric Surgery

**DOI:** 10.3389/fendo.2021.714173

**Published:** 2021-08-12

**Authors:** Marta Borges-Canha, João Sérgio Neves, Fernando Mendonça, Maria Manuel Silva, Cláudia Costa, Pedro M. Cabral, Vanessa Guerreiro, Rita Lourenço, Patrícia Meira, Daniela Salazar, Maria João Ferreira, Jorge Pedro, Ebrahim Barkoudah, Ana Sande, Eva Lau, Selma B. Souto, John Preto, Paula Freitas, Davide Carvalho

**Affiliations:** ^1^Serviço de Endocrinologia, Diabetes e Metabolismo do Centro Hospitalar Universitário de São João, Porto, Portugal; ^2^Departamento de Cirurgia e Fisiologia, Faculdade de Medicina da Universidade do Porto, Porto, Portugal; ^3^Serviço de Endocrinologia do Instituto Português de Oncologia do Porto, Francisco Gentil, EPE, Porto, Portugal; ^4^Serviço de Patologia Clínica do Centro Hospitalar Universitário Cova da Beira, EPE, Covilhã, Portugal; ^5^Faculdade de Ciências da Nutrição e Alimentação da Universidade do Porto, Porto, Portugal; ^6^Department of Medicine, Brigham and Women’s Hospital and Harvard Medical School, Boston, MA, United States; ^7^Serviço de Cirurgia Geral do Centro Hospitalar Universitário de São João, Porto, Portugal; ^8^Investigação e Inovação em Saúde (i3s), Faculdade de Medicina da Universidade do Porto, Porto, Portugal

**Keywords:** beta cell function, insulin resistance, bariatric surgery, morbid obesity, weight loss

## Abstract

**Background:**

Obesity is a multifactorial disease, which is strongly associated to other metabolic disorders. Bariatric surgery is the most effective treatment of morbid obesity. The role of beta cell function in weight loss after bariatric surgery is uncertain.

**Aim:**

To evaluate the association between beta cell function and percentage of total body weight loss (TBWL%) 1, 2, 3, and 4 years after bariatric surgery in patients with morbid obesity.

**Methods:**

Retrospective longitudinal study in patients with morbid obesity followed in our center between January 2010 and July 2018. Patients were excluded if they had diabetes at baseline or missing data on the needed parameters. We evaluated baseline Homeostatic Model Assessment of IR, Homeostatic Model Assessment of β-cell function (HOMA-beta), Quantitative Insulin Sensitivity Check Index, and Matsuda and DeFronzo index, and TBWL% at years 1 to 4. Linear regression models were used to evaluate the association of indexes of insulin resistance with TBWL% (unadjusted and adjusted for age, sex, BMI, and type of surgery).

**Results:**

There were 1,561 patients included in this analysis. HOMA-beta was negatively associated with TBWL% at second, third, and fourth years post-surgery (β = −1.04 [−1.82 to −0.26], p<0.01; β = −1.16 [−2.13 to −0.19], p=0.02; β = −1.29 [−2.64 to 0.06], p=0.061, respectively). This was not observed in the first year post-surgery nor for the other indexes. Glycemia at baseline was positively associated to EWL% at second and third years post-surgery.

**Conclusion:**

β-cell function at baseline seems to be associated to long-term weight loss, explicitly after the first year post bariatric surgery. This might be a helpful predictor of weight loss in clinical practice.

## Introduction

Obesity is currently a major health and socioeconomic concern, with a growing prevalence worldwide (1). This chronic disease is defined by the World Health Organization as an excess of body fat accumulation that can be harmful (2). Obesity is a multifactorial disease that is in the spotlight of research; however, there is still a lot to uncover (3).

Bariatric surgery is considered the most effective treatment of morbid obesity concerning the body weight loss, improvement of comorbidities, and quality of life (4). However, there is no consensual definition for either surgical success or failure (1, 5, 6). The weight loss after the procedure is thought to be influenced by a myriad of factors, both regarding the care team, the patient, and the evolving environment (1, 7). Some factors have been studied and described, such as patients’ physiological factors, age of onset, and years of obesity and pre-operative comorbidities (1, 2, 8–11).

The particular role of beta cell function and insulin resistance (IR) in weight loss after bariatric surgery is highly unknown. On the other hand, it is well established that obesity is a risk factor for IR. The positive energetic balance that is characteristic of obesity leads to an increased deposition of fat in non-adipose tissues and the deleterious effect of fat tissue accumulation on glucose metabolism is known as lipotoxicity. This effect has both been implicated in IR and pancreatic beta cells dysfunction (12). If there is a bidirectional relation and if those play a role in the weight loss after bariatric surgery is yet unknown. For instance, Parri et al. and Shantavasinkul et al. found no difference concerning weight loss in patients with and without diabetes (13, 14). Notwithstanding, Nielsen et al. found that patients without T2DM lost more weight after gastric bypass surgery (15). Also, Souteiro et al showed that patients with complete diabetes remission after bariatric surgery lost more weight (16). Contrarily, Casas-Tapia et al. recently described an association of higher HOMA-IR with increased excess weight loss after vertical gastrectomy in 91 patients (17). Further evidence from Diedisheim et al. is in agreement with this hypothesis (18). These differences might be due at least in part to the different populations studied, small number of patients included, and short time of follow up. Studies with larger populations and longer follow-up periods are lacking.

We aimed to evaluate the association between beta cell function and IR at baseline and weight loss during the first 4 years after bariatric surgery.

## Methods

This study was reviewed and approved by the ethical committee of our institution. Written informed consent for participation was not required for this study in accordance with the national legislation and the institutional requirements. Privacy of the patients included was preserved along this study. This article was written following the STROBE guidelines (19).

### Study Design

We performed a retrospective observational study evaluating all the patients with morbid obesity submitted to bariatric surgery in our tertiary centre, between January 2010 and July 2018. We have a multidisciplinary team for the treatment of severe obesity (composed by a nuclear team of endocrinologists, general surgeons, nutritionists, psychiatrists and psychologists, gastroenterologists, and anaesthesiologists). During the standard of care, patients were initially evaluated at a multidisciplinary appointment, after which the team met and reached a treatment proposal for each individual patient, including the type of surgery [laparoscopic adjustable gastric band (LAGB), Roux-en-Y gastric bypass (RYGB), or sleeve gastrectomy (GS)]. All procedures were performed according to standard technique.

### Study Participants

Patients with diabetes at baseline were excluded from the analyses (n=872). Patients with missing data on the needed parameters (stated below) were also excluded (n=162). After applying the former exclusion criteria, 1,561 patients were included in this analysis, from the 2,595 patients submitted to bariatric surgery in our institution during the study period.

### Clinical and Biochemical Parameters Evaluated

The following parameters were evaluated (collected from the medical records and biochemical results available): age, gender, weight, body mass index (BMI), waist circumference, history of diabetes, dyslipidemia and hypertension, and the type of bariatric surgery performed.

Diabetes was defined by fasting plasma glucose ≥126 mg/dl, glycated hemoglobin ≥6.5%, 2 h plasma glucose after a 75-g oral glucose tolerance test ≥200 mg/dl (20), or the use of antihyperglycemic drugs. Hypertension was defined as systolic blood pressure ≥140 mm Hg, diastolic blood pressure ≥90 mm Hg (21) or the use of antihypertensive drugs. Dyslipidemia was defined by the use of lipid-lowering agents, serum low-density lipoprotein (LDL) cholesterol ≥160 mg/dl, serum high-density lipoprotein (HDL) cholesterol <40 mg/dl, or serum triglycerides ≥ 200 mg/dl (22).

Euglycemic hyperinsulinemic clamp is considered the gold standard method to assess insulin sensitivity. Because of its complexity, it is rarely performed in the clinical setting, and several indexes have been described as simple alternatives to its use. In this study, to evaluate beta cell function and IR at baseline, we used the following pre-surgical indexes: Homeostatic Model Assessment of Insulin Resistance, HOMA-IR (nicely correlated with euglycemic hyperinsulinemic clamp); Homeostatic Model Assessment of β-cell function, HOMA-beta (complementary to the former index, which evaluates β-cell function); Quantitative Insulin Sensitivity Check Index, QUICKI (which, like HOMA-IR, evaluates insulin sensitivity); Matsuda and DeFronzo index (that also evaluates insulin sensitivity, highly correlated with euglycemic hyperinsulinemic clamp); and Disposition Index (which is essentially a measure of the functionality of the pancreas in the intact individual and that predicts the onset of type 2 diabetes) (23–26). These are built based on the following formulas:

1. HOMA-IR (27): (fasting glucose (mg/dl) × fasting insulin (μU/ml))/405.
2. HOMA-beta (28): (360 × fasting insulin (μU/ml)) / (fasting glucose (mg/dl) − 63).
3. QUICKI (28): 1/(log(fasting insulin (μU/ml)) + log(fasting glucose (mg/dl))).
4. Matsuda and DeFronzo Index (29): (10,000/square root of [fasting glucose × fasting insulin] × [mean glucose × mean insulin during oral glucose tolerance test]).
5. Disposition Index (DI) (30): Insulinogenic index × Insulin sensitivity index = [(insulin at 30 min during oral glucose tolerance test − fasting insulin) / (glucose at 30 min during oral glucose tolerance test − fasting glucose)] × [10,000/square root of (fasting glucose × fasting insulin) × (mean glucose × mean insulin during oral glucose tolerance test)].

### Outcomes and Statistical Analysis

We hypothesized that patients with worst beta cell function and/or higher levels of insulin resistance achieve less weight loss after bariatric surgery. Therefore, our main outcomes were total body weight loss (TBWL%), at 1, 2, 3, and 4 years after bariatric surgery. We used the following formula to calculate TBWL%: (weight at baseline evaluation − weight at first/ second/ third/ fourth year evaluation) / weight at baseline evaluation × 100.

We used linear regression models (unadjusted and adjusted) to evaluate the association of baseline insulin resistance indexes (log-transformed), fasting plasma glucose, HbA1c and glucose, insulin, and C Peptide at 60 and 120 min at oral glucose tolerance test, with TBWL% at first, second, third, and fourth years. The adjusted model included age, sex, BMI, and type of surgery. We also performed a sensitivity analysis excluding patients that initiated anti-diabetic medication across de follow-up time.

Continuous variables are described as mean ± standard deviation or median (25th to 75th percentiles) and categorical variables as proportions (percentages). Statistical analyses were performed with Stata software, version 14.1 (StataCorp). We considered a two-sided P value less than 0.05 to be statistically significant.

## Results

### Baseline Population Characteristics

Clinical and demographic characteristics of the population included in this analysis are presented in **Table 1**. From the 1561 individuals, 87.4% were female. The average age of the population was 40.7±10.3 years and BMI was 43.5±5.3 kg/m^2^. Thirty-five percent of the patients had dyslipidemia and 54% hypertension. Mean glycated hemoglobin was 5.5±0.4%.

**Table 1:** Clinical and demographic characteristics of the population included (n=1561).

Age, years	40.7 ± 10.3
Female, %	87.4
Weight, kg	115.0 ± 17.9
Body mass index, kg/m^2^	43.5 ± 5.3
Waist circumference, cm	121.5 ± 12.9
Dyslipidemia, %	36.1
Hypertension, %	54.0
Glycated hemoglobin, %	5.5 ± 0.4
Insulin, µU/ml	19.6 ± 13.6
C peptide, ng/mlL	3.7 ± 1.3
HOMA-IR	3.9 [2.5 – 5.7]
HOMA-beta	217.5 [144.0 – 316.6]
Matsuda and DeFronzo Index	2.5 [1.7 – 3.8]
QUICKI	0.1 [0.1 – 0.1]
Type of surgery, %	
Gastric band	10.7
Gastric bypass	58.9
Gastric sleeve	30.4

Values are shown as mean ± standard deviation or as median [percentile 25 – percentile 75]. HOMA-IR, homeostatic model assessment of insulin resistance; HOMA-beta, homeostatic model assessment of β-cell function; QUICKI, quantitative insulin sensitivity check index.

**Figure 1:**
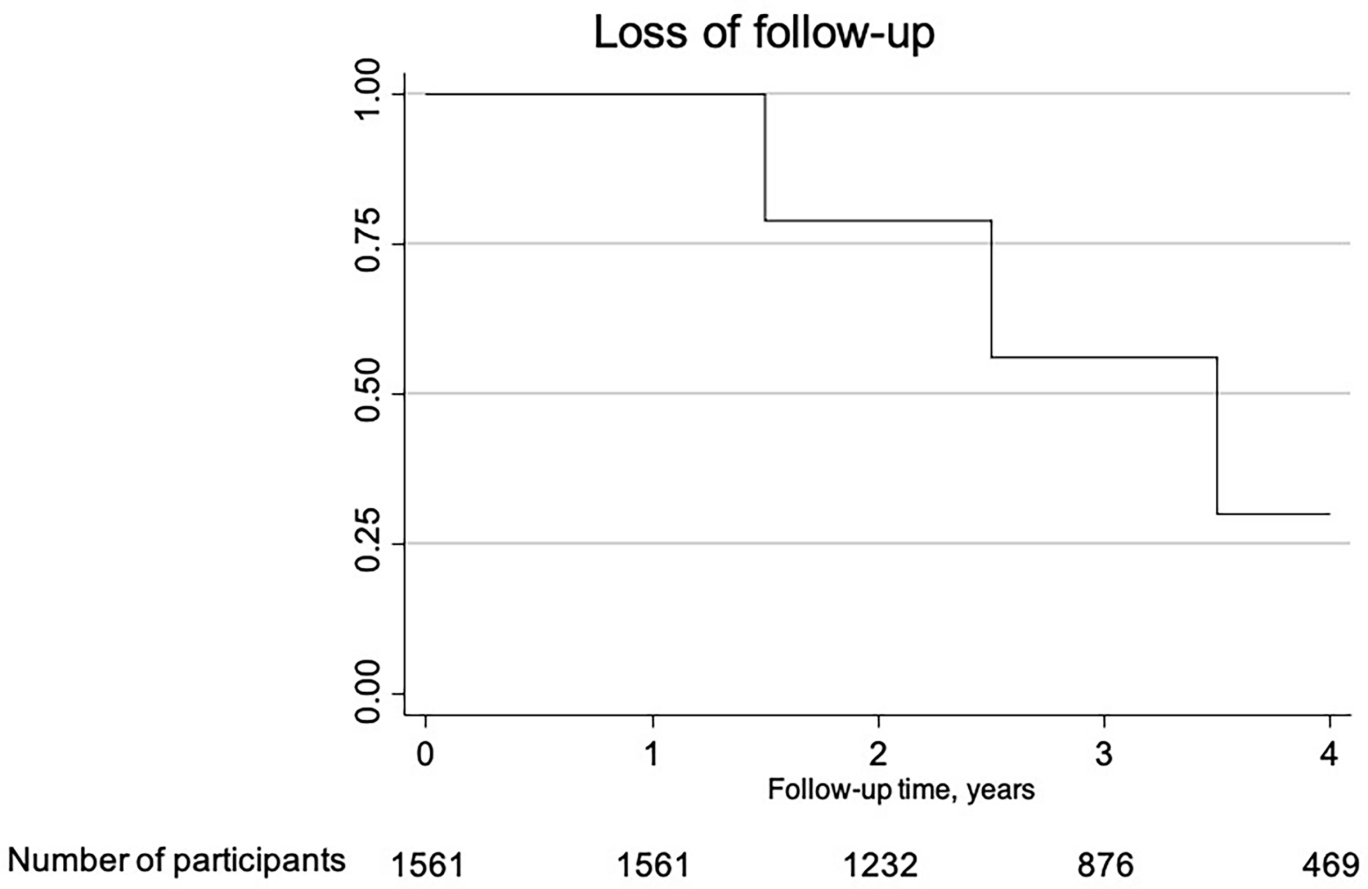
Kaplan-Meier curve representative of the lost to follow-up along the time of the study.

Concerning missing data, **Figure 1** represents the loss to follow-up across the observational time. At year 2, there were 1,232 patients remaining, 876 at the third year, and 469 at the fourth.

### Weight Loss Over Time

**Figure 2:**
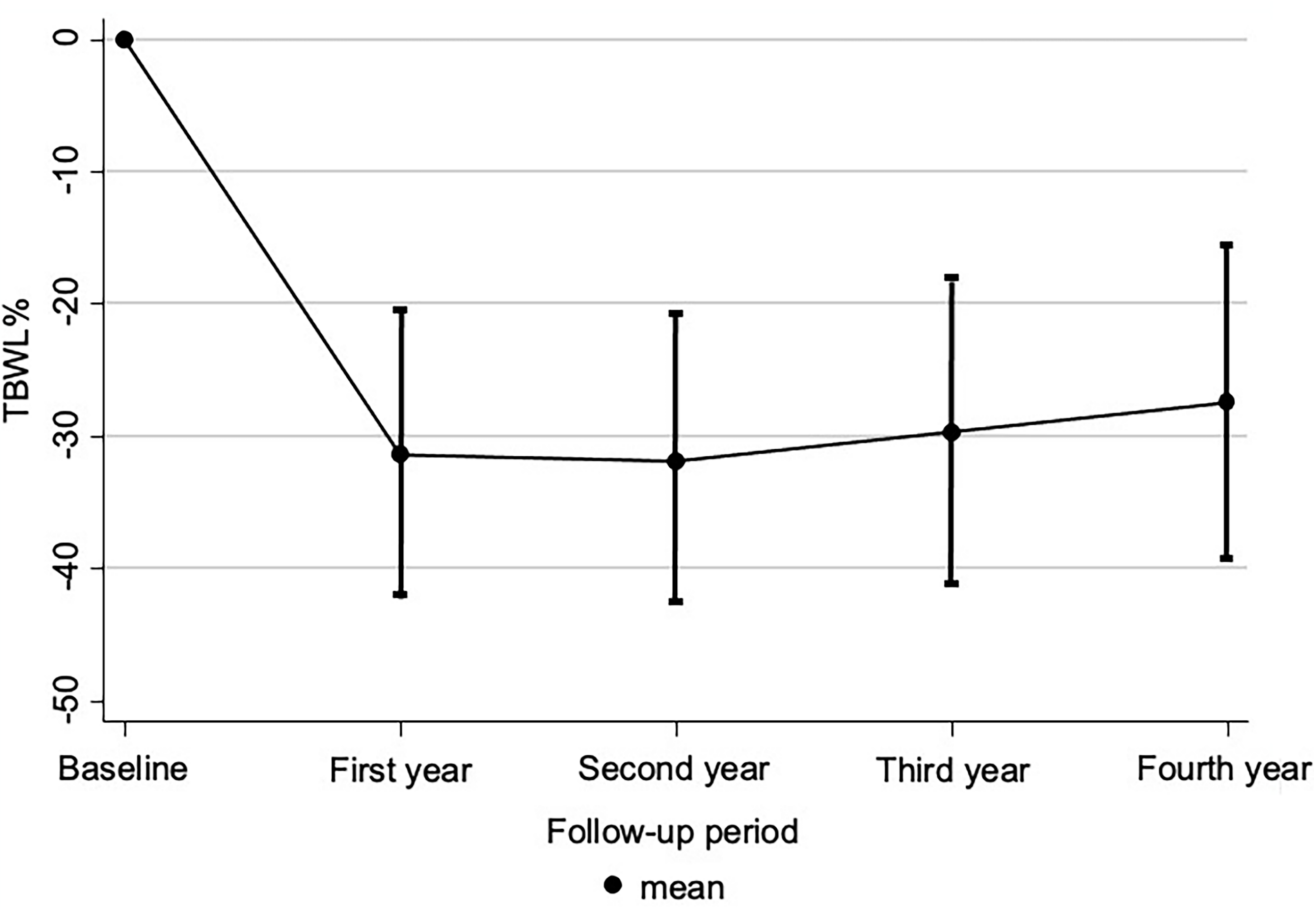
Weight plot over time. Values are show as the mean values of total body weight loss, TBWL%, (represented by the dots), and its standard deviation (vertical lines). TBWL%: − (weight at baseline evaluation − weight at first/second/third/fourth year evaluation)/weight at baseline evaluation × 100.

**Figure 2** is a smooth plot showing the TBWL% over time. The most pronounced weight loss was during the first year post-surgery (mean of weight loss of 31.2 ± 10.7 kg). From year 1 to the fourth year post-surgery, there is a trend to weight regain. The mean of weight loss was 31.6±10.9 kg, 29.6±11.6 kg, and 27.4 ±11.9 kg at the second, third, and fourth years, respectively. As expected, there is a positive correlation between BMI and weight loss and a negative correlation between age and weight loss, meaning that younger patients and patients with higher BMI tend to lose more weight (data not shown).

### Association Between TBWL% and Beta Cell Function and IR Indexes at Baseline

**Table 2** summarizes the associations between TBWL% and IR and beta cell function indexes at baseline. HOMA-beta index at baseline was negatively correlated with TBWL at the second and third years post-surgery in the adjusted analysis surgery (β = −1.04 [−1.82 to −0.26], p<0.01; β = −1.16 [−2.13 to −0.19], p=0.02, respectively), and there was a tendency for negative association in the fourth year, tough not significant. There is no correlation for the first year post-surgery.

**Table 2:** Associations between TBWL% and IR and beta cell function indexes at baseline.

	HOMA-beta		HOMA-IR		Matsuda&DeFronzo		QUICKI		Disposition Index		
	β (95% CI)	P Value	β (95% CI)	P Value	β (95% CI)	P Value	β (95% CI)	P Value	β (95% CI)	P Value
TBWL% at year 1										
Unadjusted	0.62 (−0.09 to 1.33)	0.088	0.42 (−0.26 to 1.09)	0.224	−0.16 (−1.08 to 0.76)	0.735	−2.26 (−6.40 to 1.88)	0.285	0.04 (−0.05 to 0.14)	0.356
Model 1^a^	−0.53 (−1.16 to 0.10)	0.100	−0.29 (−0.87 to 0.28)	0.317	0.33 (−0.43 to 1.09)	0.398	1.38 (−2.15 to 4.92)	0.442	−0.02 (−0.10 to 0.05)	0.544
TBWL% at year 2										
Unadjusted	0.10 (−0.78 to 0.99)	0.818	0.41 (−0.43 to 1.25)	0.343	0.03 (−1.10 to 1.15)	0.965	−2.11 (−7.34 to 3.11)	0.428	0.06 (−0.06 to 0.11)	0.326
Model 1^a^	−1.04 (−1.82 to −0.26)	**0.009**	−0.33 (−1.06 to 0.39)	0.363	0.58 (−0.36 to 1.52)	0.224	1.96 (−2.51 to 6.42)	0.390	−0.01 (−0.11 to 0.10)	0.903
TBWL% at year 3										
Unadjusted	−0.12 (−1.22 to 0.98)	0.825	0.32 (−0.71 to 1.35)	0.542	−0.10 (−1.53 to 1.33)	0.890	−0.89 (−7.20 to 5.42)	0.782	0.07 (−0.09 to 0.23)	0.366
Model 1^a^	−1.16 (−2.13 to −0.19)	**0.019**	−0.28 (−1.17 to 0.61)	0.543	0.33 (−0.89 to 1.54)	0.599	2.11 (−3.31 to 7.53)	0.445	0.00 (−0.13 to 0.14)	0.951
TBWL% at year 4										
Unadjusted	0.04 (−1.45 to 1.53)	0.958	0.04 (−1.36 to 1.44)	0.956	−0.28 (−2.16 to 1.61)	0.775	0.25 (−8.43 to 8.92)	0.955	0.15 (−0.03 to 0.33)	0.093
Model 1^a^	−1.29 (−2.64 to 0.06)	0.061	−0.57 (−1.80 to 0.66)	0.364	0.16 (−1.51 to 1.83)	0.849	3.69 (−3.94 to 11.33)	0.342	0.07 (−0.09 to 0.22)	0.392

HOMA-IR, homeostatic model assessment of insulin resistance; HOMA-beta, homeostatic model assessment of β-cell function; QUICKI, quantitative insulin sensitivity check index. The indexes were log-transformed. ^a^Adjusted for age, sex, BMI, and type of surgery. Bolded text indicates p values less than 0.05.

**Table 3** presents the associations between TBWL% and fasting glucose and glycated haemoglobin at baseline. Glycemia at baseline was positively associated with TBWL% at second and third years post-surgery.

**Table 3:** Associations between TBWL% and fasting plasma glucose and glycated hemoglobin (HbA1c) at baseline.

	Fasting plasma or blood glucose at baseline		HbA1c at baseline	
	β (95% CI)	P Value	β (95% CI)	P Value
TBWL% at year 1				
Unadjusted	−0.02 (−0.07 to 0.03)	0.479	−1.47 (−2.96 to 0.01)	0.052
Model 1^a^	0.02 (−0.03 to 0.07)	0.416	0.50 (−0.84 to 1.84)	0.463
TBWL% at year 2				
Unadjusted	0.04 (−0.02 to 0.11)	0.168	−1.09 (−2.83 to 0.64)	0.217
Model 1^a^	0.08 (0.03 to 0.14)	**0.003**	0.05 (−1.50 to 1.60)	0.951
TBWL% at year 3				
Unadjusted	0.04 (-0.04 to 0.12)	0.302	−1.37 (−3.57 to 0.84)	0.225
Model 1^a^	0.09 (0.02 to 0.16)	**0.010**	−0.06 (−2.04 to 1.92)	0.955
TBWL% at year 4				
Unadjusted	0.02 (−0.08 to 0.13)	0.659	−0.28 (−3.16 to 2.61)	0.850
Model 1^a^	0.07 (−0.02 to 0.16)	0.149	1.47 (−1.13 to 4.08)	0.266

a Adjusted for age, sex, BMI, and type of surgery.

The significance of bolded text indicates p values less than 0.05.

**Supplementary Tables 1 **and** 2** shows the sensitivity analysis performed excluding patients that initiated anti-diabetic drugs along the follow-up period. The results are overlapping with the main results. **Supplementary Tables 3A–C** show the analysis between glucose, insulin, and C Peptide at 60 and 120 min at oral glucose tolerance test, with TBWL% across the follow up.

## Discussion

This retrospective longitudinal study in patients with morbid obesity that underwent metabolic surgery aimed to evaluate the association between beta cell function and IR at the time of surgery and the weight loss at the long-term. Here, we show that the index HOMA-beta was negatively associated with TBWL% in the follow-up at second and third years after surgery. This was not observed in the first year post-surgery nor for the other indexes. Also, glycemia at baseline was positively associated to TBWL% at second and third years post-surgery.

HOMA-beta is an index, which evaluates beta-cell function and, therefore, the insulin secretory function. Lower levels of this index indicate compromised beta-cells function. On the other hand, higher fasting glycemia indicates insulin resistance and/or compromised beta-cell function (28, 31).

Our results suggest that patients with morbid obesity and worst insulin secretory function and higher fasting glycemia tend to lose more weight after bariatric surgery. Both the negative association between HOMA-beta at baseline and TBWL%, and the positive association of glycemia at baseline and TBWL% are significant at years 2 and 3, and there is also a tendency at year 4. The fact that the results are non-significant for the fourth year of follow-up may be explained by missing data due to loss to follow-up, which decreases the power for finding any significant difference. Despite not being in accordance with some studies in this area (13–15), our results are in accordance with the results described by Casas-Tapia et al. at 1 year post-vertical gastrectomy (17). This might be due to the anabolic role of insulin (32). It is biologically plausible that patients with lower production of insulin in the post-operative period may lose more weight owing to the lack of insulin anabolic role. Also, these patients are more probably the ones that were initiated on metformin along the follow-up, which is associated to weight loss, and this might be confounding our results; however, our sensitivity analyses attenuates this limitation. Also, because no other significant results were found besides the ones stated, we believe there is still a lot to uncover. Ultimately, we can state that patients with worst glycemic profiles do not seem to lose less weight that the ones with better glycemic profiles.

Concerning the weight-loss over time, our results are according to the widely known phenomena of the majority of the weight loss happening during the first year post-surgery (33, 34). This loss may be more random and due to a myriad of factors and this might explain the lack of significant differences at year 1 post-surgery (2, 5, 6).

This is a retrospective study that includes a sample of a great size of an often-overlooked population and that opens a door in this area of knowledge. There are limitations inherent to this work that must be acknowledged. First, this is a retrospective study and, as such, is more prone to specific types of bias. For instance, we have considerable missing data due to loss to follow-up, and there may be selection bias affecting our results. Also, there are possible confounders to our results that we may have not accounted with; however, we believe that our model of adjustment includes the most relevant potential confounders. Furthermore, we do not have mortality data and, therefore, survival bias is another potential confounder. Still, we believe that the importance of our results, as well as the number of individuals, included fairly overcome these limitations.

Concluding, beta cell function at baseline seems to be associated to greater long-term weight loss post bariatric surgery. Longitudinal prospective studies focusing explicitly on this research question are needed to corroborate and explain these results. Beta cell function at baseline might be a helpful predictor of weight loss in clinical practice.

## Data Availability Statement

The raw data supporting the conclusions of this article will be made available by the authors, without undue reservation.

## Ethics Statement

The studies involving human participants were reviewed and approved by Comissão de Ética do Centro Hospitalar Universitário de São João. Written informed consent for participation was not required for this study in accordance with the national legislation and the institutional requirements.

## Author Contributions

MB-C, JN, and EB contributed to the conception and design of this work, the statistical analysis, and interpretation of data. FM, MS, CC, PC, VG, RL, PM, DS, MF, JP, AS, EL, SS, JP, PF, and DC contributed to interpretation of data. MB-C prepared the first draft of this paper. All authors contributed to the article and approved the submitted version.

## Conflict of Interest

The authors declare that the research was conducted in the absence of any commercial or financial relationships that could be construed as a potential conflict of interest.

## Publisher’s Note

All claims expressed in this article are solely those of the authors and do not necessarily represent those of their affiliated organizations, or those of the publisher, the editors and the reviewers. Any product that may be evaluated in this article, or claim that may be made by its manufacturer, is not guaranteed or endorsed by the publisher.
