# Supplementary material for: Beta Cell Function as a Baseline Predictor of Weight Loss After Bariatric Surgery
Source: Front Endocrinol (Lausanne). 2021 Aug 12;12:714173. doi: 10.3389/fendo.2021.714173 (PMC8387593; doi:10.3389/fendo.2021.714173)
Supplement: Supplementary file 1 [file Table_1.docx]

| **Supplementary Table 1- Associations between TBWL% and insulin resistance indexes, excluding patients that initiated anti-diabetic drugs along the follow-up period.** | | | | | | | | | |
| --- | --- | --- | --- | --- | --- | --- | --- | --- | --- |
|  | **HOMA-beta** |  | **HOMA-IR** |  | **Matsuda&DeFronzo** |  | **QUICKI** |  |  |
|  | β (95% CI) | P Value | β (95% CI) | P Value | β (95% CI) | P Value | β (95% CI) | P Value |  |
| TBWL% at year 1 |  |  |  |  |  |  |  |  |  |
| Unadjusted | 0.72 (-0.00 to 1.45) | 0.050 | 0.56 (-0.13 to 1.25) | 0.111 | -0.22 (-1.18 to 0.73) | 0.645 | -3.11 (-7.40 to 1.19) | 0.156 |  |
| Model 1^a^ | -0.44 (-1. 80 to 0.19) | 0.172 | -0.19 (-0.78 to 0.40) | 0.522 | 0.29 (-0.50 to 1.08) | 0.477 | 0.99 (-2.65 to 4.64) | 0.594 |  |
| TBWL% at year 2 |  |  |  |  |  |  |  |  |  |
| Unadjusted | 0.28 (-0.67 to 1.24) | 0.559 | 0.39 (-0.52 to 1.30) | 0.399 | 0.01 (-1.21 to 1.24) | 0.984 | -2.49 (-8.31 to 3.32) | 0.401 |  |
| Model 1^a^ | -0.96 (-1.79 to -0.12) | **0.024** | -0.41 (-1.18 to 0.37) | 0.305 | 0.60 (-0.42 to 1.62) | 0.251 | 2.47 (-2.47 to 7.42) | 0.326 |  |
| TBWL% at year 3 |  |  |  |  |  |  |  |  |  |
| Unadjusted | 0.28 (-0.98 to 1.55) | 0.662 | 0.65 (-0.56 to 1.86) | 0.290 | -0.70 (-2.37 to 0.96) | 0.406 | -3.78 (-11.44 to 3.88) | 0.333 |  |
| Model 1^a^ | -0.97 (-2.07 to 0.13) | 0.083 | -0.21 (-1.24 to 0.82) | 0.691 | 0.11 (-1.26 to 1.48) | 0.876 | -1.35 (-5.13 to 7.83) | 0.683 |  |
| TBWL% at year 4 |  |  |  |  |  |  |  |  |  |
| Unadjusted | 0.85 (-1.06 to 2.77) | 0.382 | 0.66 (-1.14 to 2.47) | 0.470 | -0.58 (-3.00 to 1.84) | 0.637 | -3.97 (-15.46 to 7.51) | 0.496 |  |
| Model 1^a^ | -0.17 (-1.91 to 1.58) | 0.852 | 0.28 (-1.29 to 1.86) | 0.722 | -0.54 (-2.61 to 1.53) | 0.608 | -1.58 (-11.56 to 8.39) | 0.755 |  |
| HOMA-IR, Homeostatic Model Assessment of Insulin Resistance; HOMA-beta, Homeostatic Model Assessment of β-cell function; QUICKI, Quantitative Insulin Sensitivity Check Index. The indexes were log-transformed.  ^a^Adjusted for age, sex, BMI and type of surgery.  The significance of bolded text indicates p values less than 0.05. | | | | | | | | |  |

| **Supplementary Table 2- Associations between TBWL% and fasting glucose and glycated hemoglobin (HbA1c) at baseline, excluding patients that initiated anti-diabetic drugs along the follow-up period.** | | | | |
| --- | --- | --- | --- | --- |
|  | Fasting blood glucose at baseline |  | HbA1c at baseline |  |
|  | β (95% CI) | P Value | β (95% CI) | P Value |
| TBWL% at year 1 |  |  |  |  |
| Unadjusted | -0.01 (-0.07 to 0.04) | 0.615 | -1.28 (-2.77 to 0.22) | 0.094 |
| Model 1^a^ | 0.02 (-0.02 to 0.07) | 0.340 | 0.62 (-0.72 to 1.96) | 0.362 |
| TBWL% at year 2 |  |  |  |  |
| Unadjusted | 0.02 (-0.04 to 0.09) | 0.463 | -1.61 (-3.46 to 0.24) | 0.087 |
| Model 1^a^ | 0.07 (0.01 to 0.12) | **0.025** | -0.37 (-2.00 to 1.26) | 0.653 |
| TWL% at year 3 |  |  |  |  |
| Unadjusted | 0.04 (-0.05 to 0.13) | 0.362 | -1.46 (-3.91 to 0.99) | 0.242 |
| Model 1^a^ | 0.09 (0.01 to 0.17) | **0.032** | -0.40 (-2.56 to 1.76) | 0.717 |
| TBWL% at year 4 |  |  |  |  |
| Unadjusted | 0.01 (-0.12 to 0.14) | 0.878 | -0.25 (-3.85 to 3.34) | 0.891 |
| Model 1^a^ | 0.05 (-0.06 to 0.17) | 0.373 | 0.35 (-2.90 to 3.61) | 0.831 |
| ^a^Adjusted for age, sex, BMI and type of surgery.  The significance of bolded text indicates p values less than 0.05. | | | | |

| **Supplementary Table 3a- Associations between TBWL% and glucose at 60 and 120 minutes at oral glucose tolerance test (OGTT) at baseline.** | | | | |
| --- | --- | --- | --- | --- |
|  | Glucose at 60 minutes at OOGT | | Glucose at 120 minutes at OOGT | |
|  | β (95% CI) | P Value | β (95% CI) | P Value |
| TBWL% at year 1 |  |  |  |  |
| Unadjusted | -0.02 (-0.04 to 0.00) | **0.036** | -0.03 (-0.05 to -0.01) | **0.010** |
| Model 1^a^ | -0.00 (-0.02 to 0.01) | 0.618 | -0.01 (-0.03 to 0.00) | 0.150 |
| TBWL% at year 2 |  |  |  |  |
| Unadjusted | -0.01 (-0.03 to 0.01) | 0.335 | -0.03 (-0.05 to -0.00) | **0.043** |
| Model 1^a^ | -0.00 (-0.02 to 0.02) | 0.871 | -0.02 (-0.04 to 0.01) | 0.157 |
| TBWL% at year 3 |  |  |  |  |
| Unadjusted | -0.00 (-0.03 to 0.02) | 0.777 | -0.03 (-0.07 to -0.00) | **0.045** |
| Model 1^a^ | 0.01 (-0.02 to 0.03) | 0.629 | -0.01 (-0.04 to 0.02) | 0.448 |
| TBWL% at year 4 |  |  |  |  |
| Unadjusted | -0.00 (-0.04 to 0.03) | 0.833 | -0.04 (-0.08 to 0.00) | 0.073 |
| Model 1^a^ | 0.01 (-0.02 to 0.04) | 0.473 | -0.02 (-0.06 to 0.01) | 0.244 |
| ^a^Adjusted for age, sex, BMI and type of surgery.  The significance of bolded text indicates p values less than 0.05. | | | | |

| **Supplementary Table 3b- Associations between TBWL% and insulin at 60 and 120 minutes at oral glucose tolerance test (OGTT) at baseline.** | | | | |
| --- | --- | --- | --- | --- |
|  | Insulin at 60 minutes at OOGT | | Insulin at 120 minutes at OOGT | |
|  | β (95% CI) | P Value | β (95% CI) | P Value |
| TBWL% at year 1 |  |  |  |  |
| Unadjusted | 0.00 (-0.01 to 0.01) | 0.707 | -0.00 (-0.01 to 0.01) | 0.606 |
| Model 1^a^ | -0.00 (-0.01 to 0.01) | 0.722 | -0.00 (-0.01 to 0.00) | 0.190 |
| TBWL% at year 2 |  |  |  |  |
| Unadjusted | 0.00 (-0.01 to 0.01) | 0.941 | -0.00 (-0.01 to 0.01) | 0.838 |
| Model 1^a^ | -0.00 (-0.01 to 0.00) | 0.515 | -0.00 (-0.01 to 0.00) | 0.316 |
| TBWL% at year 3 |  |  |  |  |
| Unadjusted | 0.00 (-0.01 to 0.02) | 0.416 | 0.00 (-0.01 to 0.01) | 0.878 |
| Model 1^a^ | 0.00 (-0.01 to 0.01) | 0.751 | -0.00 (-0.01 to 0.01) | 0.849 |
| TBWL% at year 4 |  |  |  |  |
| Unadjusted | 0.02 (-0.00 to 0.03) | 0.053 | 0.01 (-0.01 to 0.02) | 0.473 |
| Model 1^a^ | 0.01 (-0.00 to 0.02) | 0.116 | 0.00 (-0.01 to 0.02) | 0.846 |
| ^a^Adjusted for age, sex, BMI and type of surgery.  The significance of bolded text indicates p values less than 0.05. | | | | |

| **Supplementary Table 3c- Associations between TBWL% and C Peptide at 60 and 120 minutes at oral glucose tolerance test (OGTT) at baseline.** | | | | |
| --- | --- | --- | --- | --- |
|  | C Peptide at 60 minutes at OOGT | | C Peptide at 120 minutes at OOGT | |
|  | β (95% CI) | P Value | β (95% CI) | P Value |
| TBWL% at year 1 |  |  |  |  |
| Unadjusted | 0.04 (-0.13 to 0.21) | 0.652 | -0.05 (-0.21 to 0.12) | 0.560 |
| Model 1^a^ | 0.03 (-0.11 to 0.18) | 0.645 | -0.01 (-0.14 to 0.13) | 0.933 |
| TBWL% at year 2 |  |  |  |  |
| Unadjusted | -0.04 (-0. 22 to 0.17) | 0.719 | -0.05 (-0.25 to 0.14) | 0.613 |
| Model 1^a^ | -0.06 (-0.23 to 0.11) | 0.466 | -0.06 (-0.22 to 0.10) | 0.461 |
| TBWL% at year 3 |  |  |  |  |
| Unadjusted | 0.05 (-0.21 to 0.31) | 0.687 | -0.11 (-0.36 to 0.15) | 0.406 |
| Model 1^a^ | 0.04 (-1.19 to 0.26) | 0.735 | -0.05 (-0.26 to 0.17) | 0.664 |
| TBWL% at year 4 |  |  |  |  |
| Unadjusted | 0.02 (-0.14 to 0.55) | 0.248 | -0.28 (-3.16 to 2.61) | 0.850 |
| Model 1^a^ | 0.08 (-0.24 to 0.40) | 0.622 | -0.17 (-0.47 to 0.14) | 0.294 |
| ^a^Adjusted for age, sex, BMI and type of surgery.  The significance of bolded text indicates p values less than 0.05. | | | | |
